# Do Emergency Department Patients Receive a Pathological Diagnosis? A Nationally-Representative Sample

**DOI:** 10.5811/westjem.2014.12.23474

**Published:** 2015-01-07

**Authors:** Leana S. Wen, Janice A. Espinola, Joshua M. Mosowsky, Carlos A. Camargo

**Affiliations:** * George Washington University, Department of Emergency Medicine, Washington, District of Columbia; † Massachusetts General Hospital, Department of Emergency Medicine, Boston, Massachusetts; ‡ Brigham and Women’s Hospital, Department of Emergency Medicine, Boston, Massachusetts

## Abstract

**Introduction:**

Understanding the cause of patients’ symptoms often requires identifying a pathological diagnosis. A single-center study found that many patients discharged from the emergency department (ED) do not receive a pathological diagnosis. We analyzed 17 years of data from the National Hospital Ambulatory Medical Care Survey (NHAMCS) to identify the proportion of patients who received a pathological diagnosis at ED discharge. We hypothesized that many patients do not receive a pathological diagnosis, and that the proportion of pathological diagnoses increased between 1993 and 2009.

**Methods:**

Using the NHAMCS data from 1993–2009, we analyzed visits of patients age ≥18 years, discharged from the ED, who had presented with the three most common chief complaints: chest pain, abdominal pain, and headache. Discharge diagnoses were coded as symptomatic versus pathological based on a pre-defined coding system. We compared weighted annual proportions of pathological discharge diagnoses with 95% CIs and used logistic regression to test for trend.

**Results:**

Among 299,919 sampled visits, 44,742 met inclusion criteria, allowing us to estimate that there were 164 million adult ED visits presenting with the three chief complaints and then discharged home. Among these visits, the proportions with pathological discharge diagnosis were 55%, 71%, and 70% for chest pain, abdominal pain, and headache, respectively. The total proportion of those with a pathological discharge diagnosis decreased between 1993 and 2009, from 72% (95% CI, 69–75%) to 63% (95% CI, 59–66%). In the multivariable logistic regression model, those more likely to receive pathological diagnoses were females, African-American as compared to Caucasian, and self-pay patients. Those more likely to receive a symptomatic diagnosis were patients aged 30–79 years, with visits to EDs in the South or West regions, and seen by a physician in the ED.

**Conclusion:**

In this analysis of a nationally-representative database of ED visits, many patients were discharged from the ED without a pathological diagnosis that explained the likely cause of their symptoms. Despite advances in diagnostic testing, the proportion of pathological discharge diagnoses decreased. Future studies should investigate reasons for not providing a pathological diagnosis and how this may affect clinical outcomes.

## INTRODUCTION

Research into patient preferences suggests that patients value a precise diagnosis from their doctors.^1^ Understanding the diagnosis is seen to be the first step of healing, allowing for discussions of prognosis and treatment. However, anecdotal reports suggest that many patients are discharged from the emergency department (ED) without a diagnosis that explains the likely nature and cause of their symptoms. That is, these patients are discharged with the same diagnosis as their chief complaint (e.g., “chest discomfort”), rather than a specific pathological diagnosis (e.g., “gastritis”).

To our knowledge, only one study has examined the proportion of ED patients who receive symptomatic versus pathological discharge diagnoses.^2^ This pilot study was a chart review over a one-month period at a single, urban teaching hospital. As hypothesized, the authors found that most patients were discharged from the ED without a pathological diagnosis that explained the likely cause of their symptoms.

In this study, we used a national database with annually reported data from 1993–2009 to examine the proportion of ED patients who are discharged with symptomatic versus pathological discharge diagnoses. Based on the results of the single-center pilot study, we hypothesized that many patients do not receive a pathological diagnosis. Given advances in diagnostic testing, we further hypothesized that the proportion of pathological diagnoses increased between 1993 and 2009.

## METHODS

### Study Design

We conducted a cross-sectional analysis of the ED component of the 1993–2009 National Hospital Ambulatory Medical Care Survey (NHAMCS) database. NHAMCS was designed by the U.S. National Center for Health Statistics of the Centers for Disease Control and Prevention and is a national probability survey conducted for hospital outpatient and ED visits.^3^ The local institutional review board approved this study.

### Study Setting and Population

The NHAMCS is a four-stage probability sample survey gathering data from non-institutional general and short-stay hospitals in the U.S., excluding federal, military and Veteran Administration hospitals. NHAMCS is conducted annually and covers geographic primary sampling units, hospitals within primary sampling units, EDs within hospitals, and patients within EDs. The non-response rate for most items was <5%, and error rates were <2% for items requiring medical coding. National estimates were obtained through use of a multistage estimation procedure and patient visit weights.

Our study population included all ED visits by patients age ≥18 years in the 1993–2009 NHAMCS database who presented with the three most common chief complaints (as coded in NHAMCS as “reason for visit”), and who were subsequently discharged from the ED. Those three most common chief complaints were chest pain, abdominal pain, and headache. Separately, two emergency physicians coded all International Classification of Diseases-9 discharge diagnoses corresponding to these chief complaints as symptomatic or pathological diagnoses. There was 100% inter-rater agreement in the coding. Visits were categorized as a symptomatic discharge diagnosis if the discharge diagnoses (up to three per visit) contained only symptomatic and no pathological diagnosis code (e.g., “abdominal pain” alone, without specific diagnoses such as “biliary colic”). All others that contained either solely pathological diagnosis code or both symptomatic and pathological diagnoses were categorized as pathological (e.g., “biliary colic” alone, or “abdominal pain” and “biliary colic”).

### Data Analysis

We performed all analyses using Stata 11.0 (StataCorp, College Station, TX). To account for the complex 4-stage sampling frame, we performed all analyses using the survey design variables and appropriate survey commands in Stata.

We compared weighted annual proportions of pathological discharge diagnoses with 95% CIs. Annual trends in the proportion of pathological discharge diagnoses were analyzed using weighted logistic regression. Additionally, we created a multivariable logistic regression model predicting discharge with a symptomatic diagnosis, with results reported in odds ratios (OR) and 95% CIs. A two-tailed p-value <0.05 was considered statistically significant.

## RESULTS

Among the 299,919 sampled visits, 44,742 visits met inclusion criteria. From these data, we estimated that there were 164 million (95% CI, 151–178 million) adult ED visits who presented with the three most common chief complaints and who were later discharged to home. Among these ED visits, the proportions with pathological discharge diagnosis were 55% for chest pain, 71% for abdominal pain, and 70% for headache (Table 1). Between 1993 and 2009, the proportion of pathological discharge diagnoses significantly decreased among those presenting with any of these three chief complaints (p ≤ 0.02 for all; Figure).

In the multivariable logistic regression model (Table 2), those presenting with any of the chief complaints of chest pain, abdominal pain, and headache who were more likely to receive pathological diagnoses were females, African-American as compared to Caucasian, Hispanics, and self-pay patients. Patients aged 30–79 years, with visits to EDs in the South or West regions, and those seen by a physician in the ED were more likely to receive a symptomatic discharge diagnosis.

## DISCUSSION

In this major subset of a nationally-representative database of ED visits from the U.S., many patients were discharged from the ED without a pathological diagnosis that explained the likely cause of their presenting symptoms. These results are similar to those obtained from a pilot study at a single teaching hospital in Boston.^2^ Reasons for physicians choosing a symptomatic rather than pathological discharge diagnosis are varied, and include individual style (e.g. not wanting to commit to a specific diagnosis), concern of malpractice (e.g. thinking that a symptomatic diagnosis is more defensible), and billing (e.g. assuming that a higher level of billing can be justified for those with an undifferentiated diagnosis). Some physicians would further argue that obtaining a definitive, pathological diagnosis is often not possible in the ED setting and that our goal in the ED should be to “rule out” life-threatening diseases and not to make pathological diagnoses. With growing recognition of the goal of patient-centeredness, this must be weighed against the desire by many patients to receive a pathological diagnosis.^4^

The results raise several interesting questions. For example, contrary to our second hypothesis, despite advances in diagnostic testing and technology, the proportion of pathological discharge diagnoses decreased. Either the ready availability and accuracy in diagnostic testing have contributed to more unwillingness to commit to a pathological diagnosis, or practice patterns are shifting due to the other reasons listed above. In addition, we find it curious that women, ages 30–79, African-American and Hispanic patients are more likely to be provided with a pathological diagnosis, and that patients seen by physicians are more likely to be given a symptomatic diagnosis. We encourage further research in this neglected research topic to elucidate the reasons behind these variations.

This study also raises the overarching question of whether provision of a pathological diagnosis helps not just patient satisfaction but also clinical outcomes. Anecdotal reports suggest that patients are better able to understand their prognosis and treatment options if provided with a specific pathophysiologic diagnosis, and some studies have correlated unscheduled returns to the ED with medical error and with lack of patient understanding their diagnosis and prognosis.^5^–^7^ Discharge communication may be particularly important for ED patients who are discharged home and may not have ready access to follow up.^8^ Future studies can focus on the perceived importance to patients of being given a pathological diagnosis at ED discharge, as well the impact of receiving a pathological diagnosis on objective health outcomes.

### LIMITATIONS

Like all survey research, there is the possibility of error in data collection and coding, and in using a secondary data source. NHAMCS data use an ED-based sample and is not population based; thus, caution should be used regarding generalizing the results to the overall population. There are also limitations of coding symptomatic and pathological diagnoses. However, criteria for coding charts were clearly defined in advance. The strong consistency of the two reviewers’ independent coding (>99% agreement) also argues against this bias. In addition, we studied only three presenting complaints. The three we studied are the most common chief complaints in the ED. While it is possible that the many excluded chief complaints will have clear pathological diagnoses (e.g., “fracture”), at the same time, excluded complaints may also be more prone to symptomatic classifications (e.g., “weakness”). Finally, this study only examined discharge diagnoses. It is possible that verbal or written discharge instructions provided information on specific diagnoses, though results from a prior study involving chart review suggest that the proportion of diagnoses provided at discharge was low,^2^ and studies have commented on the inadequacy of the discharge communication process.^8^

## CONCLUSION

According to our analysis of a nationally-representative database of ED visits, many patients with the three most common chief complaints of chest pain, abdominal pain, and headache are discharged from the ED without a pathological diagnosis that explains the likely cause of their presenting symptoms. Despite advances in diagnostic testing and technology, the proportion of pathological discharge diagnoses decreased between 1993 and 2009. We encourage further research to identify the reasons why ED clinicians often do not provide a pathological diagnosis, and to examine whether provision of a pathological diagnosis affects patient satisfaction and clinical outcomes.

## Figures and Tables

**Figure:**
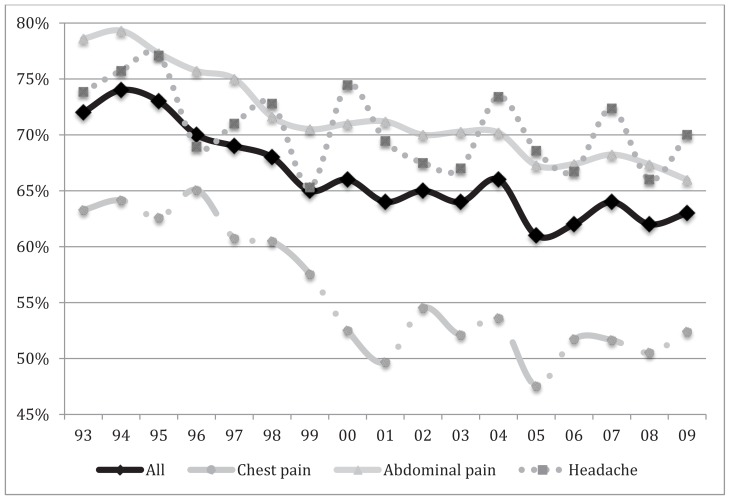
Proportion of emergency department patients discharged with pathological discharge diagnosis for three most common chief complaints, 1993–2009.

**Table 1:** Proportion of pathological discharge diagnosis for the three most common chief complaints among U.S. emergency department visits, 1993–2009.

		% (95% CI)		
		
Chief complaint	No. of visits (n)	Pathological discharge diagnosis, 1993–2009	Pathological discharge diagnosis, 1993	Pathological discharge diagnosis, 2009
Chest pain	7,666	55% (54–57%)	63% (58–69%)	52% (48–57%)
Abdominal pain	14,766	71% (70–72%)	79% (75–82%)	66% (62–70%)
Headache	7,180	70% (69–72%)	74% (70–78%)	70% (65–75%)
Any of the 3 complaints	29,612	66% (65–67%)	72% (69–75%)	63% (59–66%)

**Table 2:** Multivariable logistic regression model predicting pathological discharge from U.S. emergency departments, 1993–2009.

Characteristics	Odds ratio (95% CI)	p-value
Age		
18–29	1.00 (Reference)	
30–39	0.85 (0.80–0.91)	<0.001
40–49	0.77 (0.72–0.83)	<0.001
50–59	0.77 (0.71–0.84)	<0.001
60–69	0.84 (0.75–0.93)	0.001
70–79	0.85 (0.75–0.97)	0.02
80+	0.95 (0.82–1.10)	0.47
Sex		
Male	1.00 (Reference)	
Female	1.11 (1.05–1.17)	<0.001
Race		
White	1.00 (Reference)	
Black	0.86 (0.80–0.93)	<0.001
Other	0.90 (0.79–1.02)	0.11
Ethnicity		
Non-Hispanic	1.00 (Reference)	
Hispanic	0.85 (0.78–0.94)	0.001
Unknown	0.98 (0.91–1.06)	0.64
Insurance		
Private	1.00 (Reference)	
Public	1.02 (0.96–1.09)	0.44
Other	1.26 (1.11–1.43)	<0.001
Self-pay	1.20 (1.11–1.29)	<0.001
Unknown	1.00 (0.99–1.15)	0.96
Region		
Northwest	1.00 (Reference)	
Midwest	0.88 (0.79–0.99)	0.03
South	0.87 (0.79–0.95)	0.004
West	0.84 (0.76–0.93)	0.001
Urban		
MSA	1.00 (Reference)	
Non-MSA	1.13 (1.01–1.26)	0.03
Hospital ownership		
Voluntary non-profit	1.00 (Reference)	
Government, non-federal	1.15 (1.03–1.28)	0.010
Proprietary	1.15 (1.05–1.26)	0.003
Season of visit		
Winter (December–February)	1.00 (Reference)	
Spring (March–May)	0.97 (0.90–1.06)	0.52
Summer (June–August)	0.94 (0.86–1.02)	0.12
Fall (September–November)	0.89 (0.82–0.97)	0.01
Seen by physician	0.78 (0.65–0.94)	0.01

*MSA*, Metropolitan statistical area
